# Associations among sleep, hematologic profile, and aerobic and anerobic capacity of young swimmers: A complex network approach

**DOI:** 10.3389/fphys.2022.948422

**Published:** 2022-08-24

**Authors:** Mauricio Beitia Kraemer, Ana Luíza Paula Garbuio, Luisa Oliveira Kaneko, Claudio Alexandre Gobatto, Fúlvia Barros Manchado-Gobatto, Ivan Gustavo Masseli dos Reis, Leonardo Henrique Dalcheco Messias

**Affiliations:** ^1^ Research Group on Technology Applied to Exercise Physiology (GTAFE), Laboratory of Multidisciplinary Research, São Francisco University, Bragança Paulista, Brazil; ^2^ Laboratory of Applied Sport Physiology, School of Applied Sciences, University of Campinas, Limeira, Brazil

**Keywords:** swimming, aerobic capacity, adolescents, young athletes, critical velocity, sleep

## Abstract

Although the link between sleep and hematological parameters is well-described, it is unclear how this integration affects the swimmer’s performance. The parameters derived from the non-invasive critical velocity protocol have been extensively used to evaluate these athletes, especially the aerobic capacity (critical velocity—CV) and the anaerobic work capacity (AWC). Thus, this study applied the complex network model to verify the influence of sleep and hematological variables on the CV and AWC of young swimmers. Thirty-eight swimmers (male, *n* = 20; female, *n* = 18) completed five experimental evaluations. Initially, the athletes attended the laboratory facilities for venous blood collection, anthropometric measurements, and application of sleep questionnaires. Over the 4 subsequent days, athletes performed randomized maximal efforts on distances of 100, 200, 400, and 800-m. The aerobic and anerobic parameters were determined by linear function between distance vs. time, where CV relates to the slope of regression and AWC to *y*-intercept. Weighted but untargeted networks were generated based on significant (*p* < 0.05) correlations among variables regardless of the correlation coefficient. Betweenness and eigenvector metrics were used to highlight the more important nodes inside the complex network. Regardless of the centrality metric, basophils and red blood cells appeared as influential nodes in the networks with AWC or CV as targets. The role of other hematologic components was also revealed in these metrics, along with sleep total time. Overall, these results trigger new discussion on the influence of sleep and hematologic profile on the swimmer’s performance, and the relationships presented by this targeted complex network can be an important tool throughout the athlete’s development.

## Introduction

The link between sleep and immunity is well-established (Besedovsky et al., 2019), and poor sleep quality is associated with diseases, that, in turn, may affect hematological variables (Lippi et al., 2015; Nahrendorf and Swirski, 2015; Lee et al., 2017; Thomas and Calhoun, 2017; Zheng et al., 2018). Moreover, sleep apnea contributes to cardiovascular complications (Gabryelska et al., 2018), metabolic diseases (Light et al., 2018), and oscillations in sleep debt which have been associated with the activation of innate inflammatory pathways (Prather et al., 2009). Although the straight association between sleep and blood cell profile was described under pathologic conditions, it is unclear to what extent such interaction affects the aerobic and anerobic capacity of athletes.

The aerobic and anerobic systems are influenced by a myriad of factors, but reviews have gathered a large body of data suggesting that sleep plays an important role in these outcomes (Pilcher and Huffcutt, 1996; Fullagar et al., 2015; Watson, 2017). However, the solid fundamentals of this association remain in light of discovery, and a possible path lies within the modulation of hematological parameters caused by sleep disturbances. On the other hand, the assessment of sleep and hematological variables yields large groups of results (e.g., sleep total time, efficiency, latency, sleepiness, and red and white blood cells profile), and complex models can integrate this information to verify its impact on the aerobic and anerobic capacity inside the sports science field.

Complex networks have been applied in natural sciences and revealed relevant topological structures regarding medical purposes (Barabasi et al., 2011), metabolic changes across the human lifespan (Barajas-Martinez et al., 2020), and also concerning the sleep stages (Bashan et al., 2012). The networks operate under an untargeted shape. In this scenario, significant (*p* < 0.05) correlations among variables can be used to identify the most influential nodes in the topological structure (Gobatto et al., 2020; Cirino et al., 2021); within this analysis, every node has the same relevance inside the network. Early studies (Park et al., 2004; Li et al., 2017; Sousa et al., 2020), however, inspired also to select targets inside the topology in a pre-analytical context. Therefore, the “target nodes” have a higher weight, providing headed information within the complex network.

In the sports science field, the complex networks elucidated variables affecting the fatigue process related to physical exercise (Pereira et al., 2015) and also identify critical components for different sports modalities (Pereira et al., 2018; Ribeiro et al., 2019; Cirino et al., 2021; Breda et al., 2022), including swimming (Pereira-Ferrero et al., 2019; Fiori et al., 2022). However, the relationship between sleep and hematological variables, as well as their impact on the aerobic/anerobic capacity of young swimmers, was not explored. Both targeted and untargeted networks can reveal relevant data in this context. While the untargeted scenario can initially indicate relevant nodes in the integrated system (i.e., sleep, hematologic, and aerobic/anaerobic parameters), the target approach would confirm the impact of these nodes restrictedly to the swimmer’s metabolisms.

To advance on the aerobic and anerobic data of young swimmers, parameters accessible and robust to coaches and athletes must be considered in the complex network. Accordingly, the critical velocity protocol is a non-invasive test that provides parameters widely adopted for swimming training purposes (Toubekis et al., 2006; Zacca et al., 2010; Neiva et al., 2011; Toubekis and Tokmakidis, 2013; Zacca et al., 2016). The critical velocity (CV) was originally defined as the velocity that can be maintained without exhaustion (Wakayoshi et al., 1992a; Wakayoshi et al., 1992b; Wakayoshi et al., 1992c). During exercise whose intensity is above CV, the anerobic work capacity (AWC) represents a finite amount of work performed until exhaustion (Jones et al., 2010). Accumulated evidence has suggested that both parameters should be considered components of an integrated bioenergetic system that provides a valid framework for understanding exercise fatigue and intolerance (Poole et al., 2016). Not surprisingly, CV and AWC are sensitive to performance-enhancing manipulations and were suggested as promising doping detection (Puchowicz et al., 2018).

Overall, the CV and AWC are relevant parameters to prescribe exercise intensity and evaluate the physiological enhancement of young swimmers. By gathering and associating sleep and hematological profile with these parameters, the complex network model may reveal relevant outcomes and evidence of the interrelationships among these data. Therefore, this study aimed to apply the complex network model to verify the influence of sleep and hematological variables on the critical velocity parameters.

## Materials and methods

### Experimental design and subjects

**FIGURE 1:**
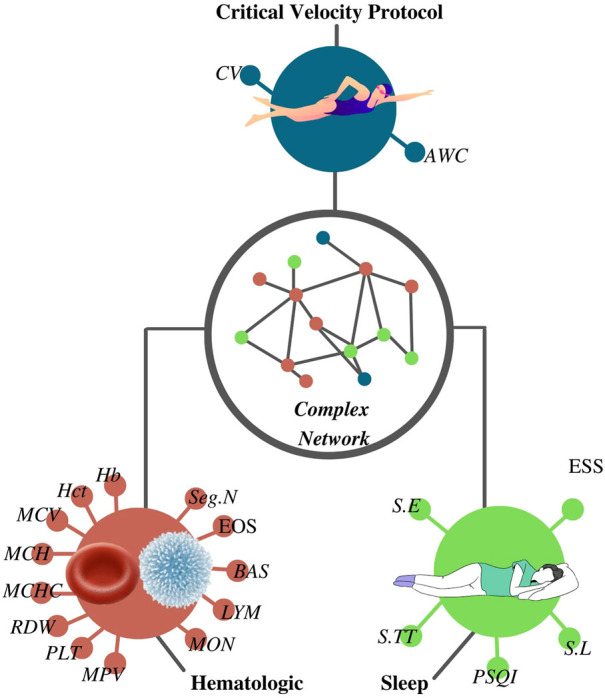
Complex network was constructed according to the results from the critical velocity protocol, sleep, and hematological analyses. CV, critical velocity; AWC, anerobic work capacity; RBC, red blood cells; Hb, hemoglobin; Hct, hematocrit; MCV, mean corpuscular volume; MCH, mean corpuscular hemoglobin; MCHC, mean corpuscular hemoglobin concentration; RDW, red cell distribution width; PLT, platelet; MPV, mean platelet volume; WBC, white blood cells; Seg.N, segmented neutrophils; EOS, eosinophils; BAS, basophils; LYM, lymphocytes; MON, monocytes; S.TT, sleep total time; S.E, sleep efficiency; S.L, sleep latency; PSQI, Pittsburgh sleep quality index score; ESS, Epworth sleepiness scale score.

This is an experimental and controlled study associating sleep and hematological variables with the parameters from the critical velocity protocol *via* the complex network model (Figure 1). Throughout the experiment, researchers instructed athletes to keep the same individual hydration/food habits. Thirty-eight young swimmers (male, *n* = 20; age = 15 ± 2 years; body mass = 61 ± 11 kg; height = 166 ± 16 cm; female, *n* = 18; age = 14 ± 2 years; body mass = 55 ± 9 kg; height = 160 ± 7 cm) completed five experimental evaluations. In the first session, the swimmers attended the laboratory facilities for venous blood collection, anthropometric measurements, and application of sleep questionnaires. Over the 4 subsequent days, athletes performed the critical velocity protocol for the determination of the aerobic and anerobic parameters. Swimmers completed the critical velocity bouts at the same time of day, 48 h apart. Young swimmers were at the beginning of the general preparatory period according to the training periodization formulated by coaches. Researchers instructed coaches to avoid having physical training during the experimental period. Therefore, athletes only performed light and recreational activities throughout critical velocity protocol.

### Critical velocity protocol

**FIGURE 2:**
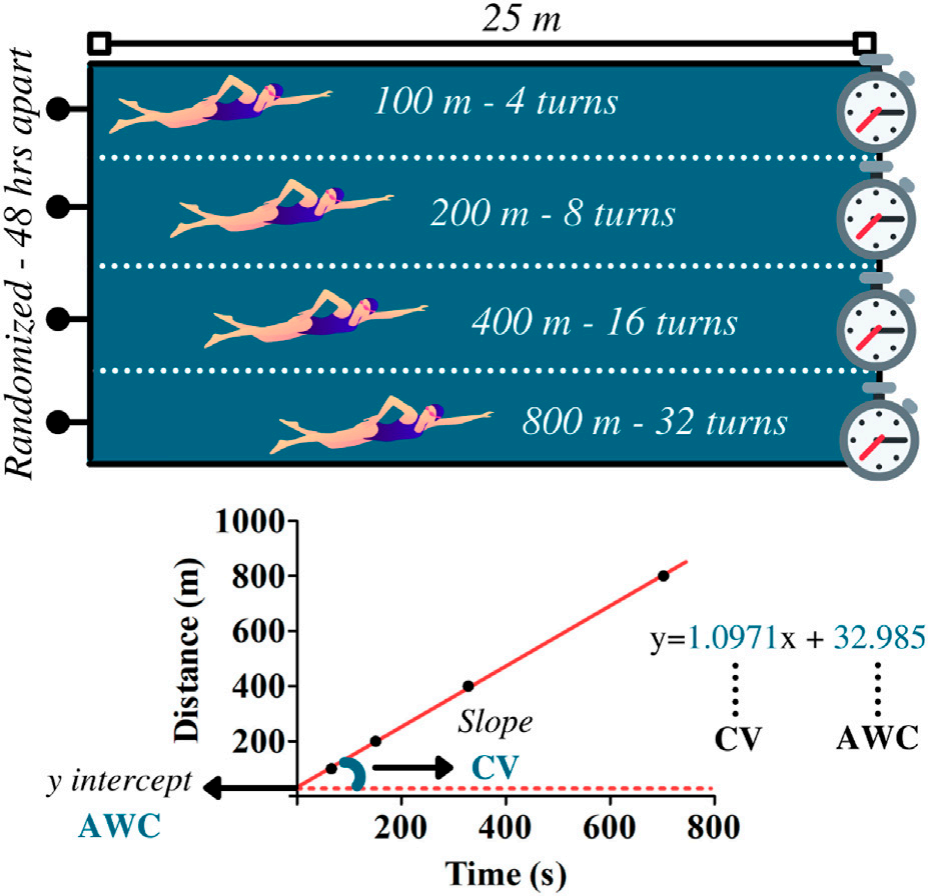
Upper panel illustrates the maximal efforts performed at 100, 200, 400, and 800 m during the critical velocity protocol. Based on the distances and the time to complete the efforts, linear regressions were constructed for the determination of the anerobic work capacity (AWC, *y*-intercept) and the critical velocity (CV, the slope of the regression). The data refer to subject 1.

Swimmers performed four randomized maximal efforts on distances of 100, 200, 400, and 800 m in a swimming pool (25-m) during the critical velocity protocol. Efforts were performed on different days at 48-h intervals (Figure 2). Researchers and coaches instructed athletes to provide their best performances throughout trials. The CV and AWC were determined by the following linear equation:

D=CV∗t + AWC. (1)

Eq. 1 Linear equation for CV and AWC determination

where D is equivalent to distance and t is related to time to cover the distance. In this model, CV relates to the slope of regression and AWC to *y*-intercept. The coefficient of determination (*R* ^2^) was used to indicate the reliability of the linear adjustment.

### Sleep analyses

The validated version (Bertolazi et al., 2011) for the Portuguese language of the Pittsburgh Sleep Quality Index (PSQI) (Buysse et al., 1989) consists of 19 questions divided into components, including sleep quality, latency, total sleep time, efficiency, disturbance, use of sleep medication, and daytime dysfunction. Each component is equally weighted on a 0–3 scale. The sum of the component scores results in the PSQI global score. Scores ≤5 represent good sleep quality, whereas scores >5 represent poor sleep quality. Researchers instructed subjects and explained PSQI questions in case of doubts. Subjects answered the PSQI in an isolated room without noise and visual interference. The Epworth Sleepiness Scale (ESS) (Johns, 1994) comprises eight questions on the usual chances of having dozed off or fallen asleep while engaged in distinct activities. Each question has a 4-point scale (0–3), and the sum of the scores provide the ESS final score. Overall, PSQI score, sleep total time (S.TT), sleep latency (S.L), sleep efficiency (S.E), and ESS score were considered sleep variables in the complex network model.

### Hematological analyses

Researchers instructed swimmers to avoid alcohol ingestion or any unusual food or beverage over 3 days before blood collection. An experienced nurse collected 5 ml of venous blood for hematological analyses. Then, samples were taken to a specialized laboratory, and the Coulter LH 750 hematology analyzer (Beckman Coulter, Miami, FL, United States) (Igout et al., 2004) assessed red blood cell (RBC) profile comprising the following parameters: hemoglobin (Hb), hematocrit (Hct), mean corpuscular volume (MCV), mean corpuscular hemoglobin (MCH), mean corpuscular hemoglobin concentration (MCHC), red cell distribution width (RDW), platelet (PLT) and mean platelet volume (MPV). Likewise, white blood cells (WBCs) like segmented neutrophils (Seg.N), eosinophils (EOS), basophils (BAS), lymphocytes (LYM), and monocytes (MON) were also determined.

### Complex network and statistical analysis

Initially, untargeted and unweighted networks were adapted from previous untargeted but weighted complex models adopted in sports sciences (Pereira et al., 2015; Pereira et al., 2018). In the present study, the criteria to obtain the network topology was similar to those used by Gobatto et al. (2020), in which only significant (*p* < 0.05) correlations among variables were considered regardless of the correlation coefficient. Thus, each variable that had an association with another one was represented in the network as a node, and the associations between nodes were represented by edges linking these nodes. In the untargeted approaches, no distinction between nodes was carried out, implying that all nodes started with the same weight and, therefore, the calculated centrality scores reflect a general systemic analysis. Then, inspired by earlier studies (Park et al., 2004; Li et al., 2017; Sousa et al., 2020), weighted and targeted complex networks were created to select targets inside the topology, that is, CV or AWC. In these approaches, both positive and inverse correlations were treated equally and received positive weights regardless of the correlation direction.

Concerning the targeted betweenness approach, two networks were assembled with the scores for the most frequent nodes in the shortest paths between the CV or AWC (targets) for all other nodes. Prior to this, the length of an edge was calculated as the difference between the highest Spearman’s correlation coefficient (1.0; constant value) and the Spearman’s correlation coefficient between the nodes connected by the edge (can vary from 0.01 to 1; higher means shorter). Thus, the edge lengths were used as the distance between nodes in the calculation of the target betweenness scores.

The target eigenvector approaches compute the centrality of a node based on the centrality of its neighbors and the weights of its edge connections. The edge weights were calculated as the product of the edge degree of proximity to the target node CV or AWC (can vary from 0.01 to 1; higher means closer) and the Spearman’s correlation coefficient between the nodes connected by the edge (can vary from 0.01 to 1; higher is better). Therefore, edges received a weight equivalent to their respective correlation coefficient when they are directly linked to the node of interest (CV or AWC). Second-degree connections with the node of interest were equivalent to 0.5 (half) of the correlation coefficients, while third-, fourth-, and fifth-degree connections were equivalent to 0.250, 0.125, and 0.0625, respectively. Thus, the edge weights were used as the connection strength in the calculation of the target eigenvector scores.

Centrality betweenness and eigenvector values were obtained utilizing a Python (release 3.9.3) application, developed specifically for the study, and NetworkX 2.5 library (Hagberg et al., 2008). The Shapiro–Wilk test verified the data as non-normal. Therefore, the correlation analysis was proceeded by the Spearman approach. Data are expressed as mean ± standard deviation. Confidence intervals were also calculated for standard deviation with *α* = 0.05 (σ/√n).

### Betweenness centrality analysis

The usual method to determine betweenness centrality in sports sciences is computing the number of shortest paths passing through some node using all nodes of the network as sources and also as targets (Pereira et al., 2015; Gobatto et al., 2020).

$$cB\left(v\right)=\;\sum_{s\in S,t\in T}\frac{\sigma \left(s,\;t/v\right)}{\sigma \left(s,\;t\right)}.$$ (2)

Eq. 2 Betweenness centrality for a subset of nodes.

In Eq. 2, S is the set of sources, T is the set of targets, σ(s,t) is the number of shortest (s,t)-paths, and σ(s,t|v) is the number of those paths passing through some node v other than s,t. If s = t, σ(s,t) = 1, and if v ∊, tσ(s,t|v) = 0 (Brandes, 2008).

### Eigenvector centrality analysis

The eigenvector centrality for node i is the *i*th element of the vector x defined by the equation where A is the adjacency matrix of the graph G with eigenvalue *λ*. There is a unique solution x, all of whose entries are positive if *λ* is the largest eigenvalue of the adjacency matrix A (Newman, 2010).

Ax=λx. (3)

Eq. 3 Eigenvector centrality for a subset of nodes.

## Results

**FIGURE 3:**
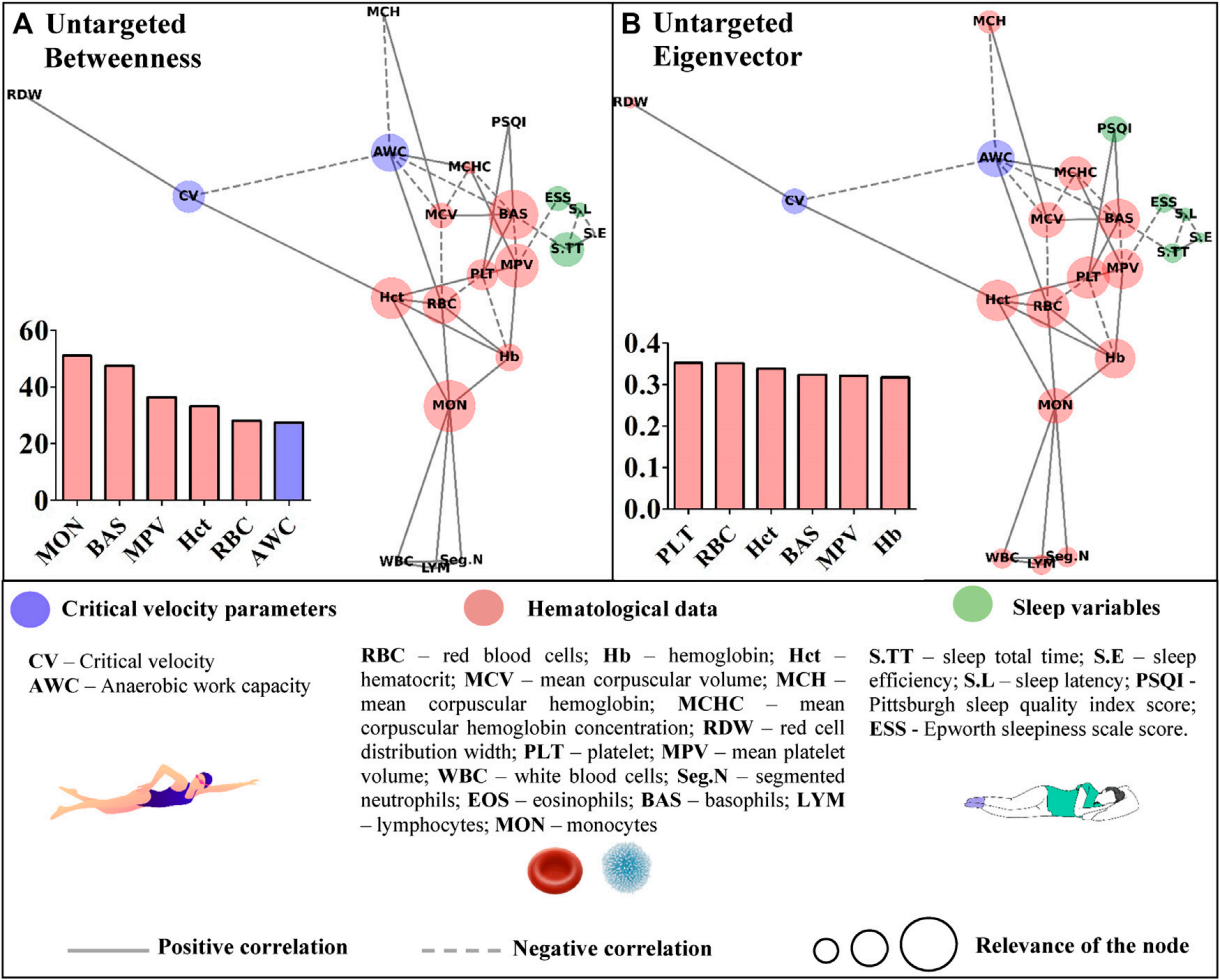
Centrality measurements from the untargeted complex network model; **(A)** betweenness analysis; **(B)** eigenvector analysis.

**FIGURE 4:**
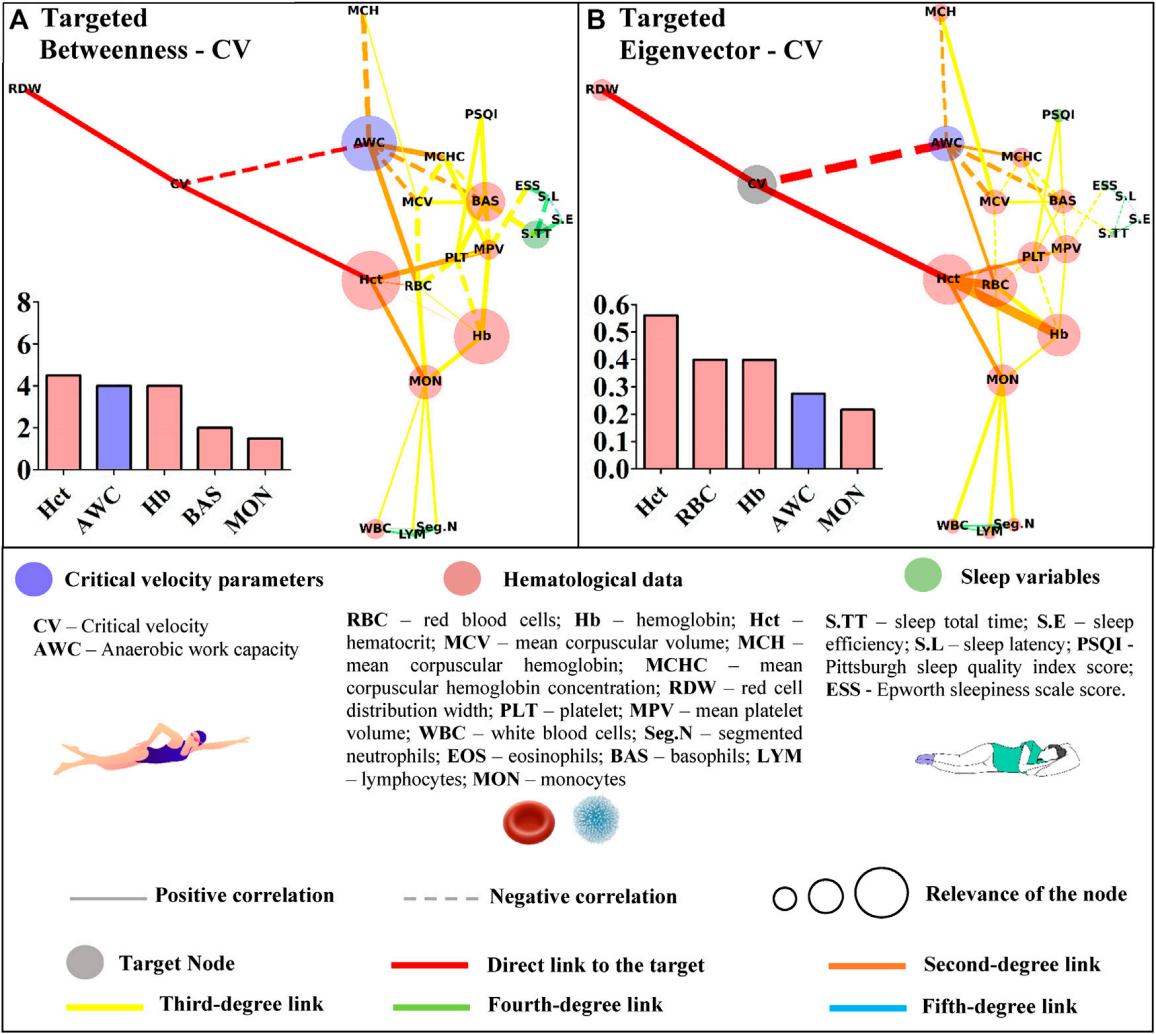
Centrality measurements from the targeted complex network model; **(A)** betweenness analysis considering the critical velocity (CV) as the target node. The thickness of the edge is directly related to the “distance” between the nodes connected by the edge (lower thickness means closer distances); **(B)** eigenvector analysis considering the critical velocity (CV) as the target node. The thickness of the edge is directly related to the strength of the edge connection (lower thickness means weaker connections).

**FIGURE 5:**
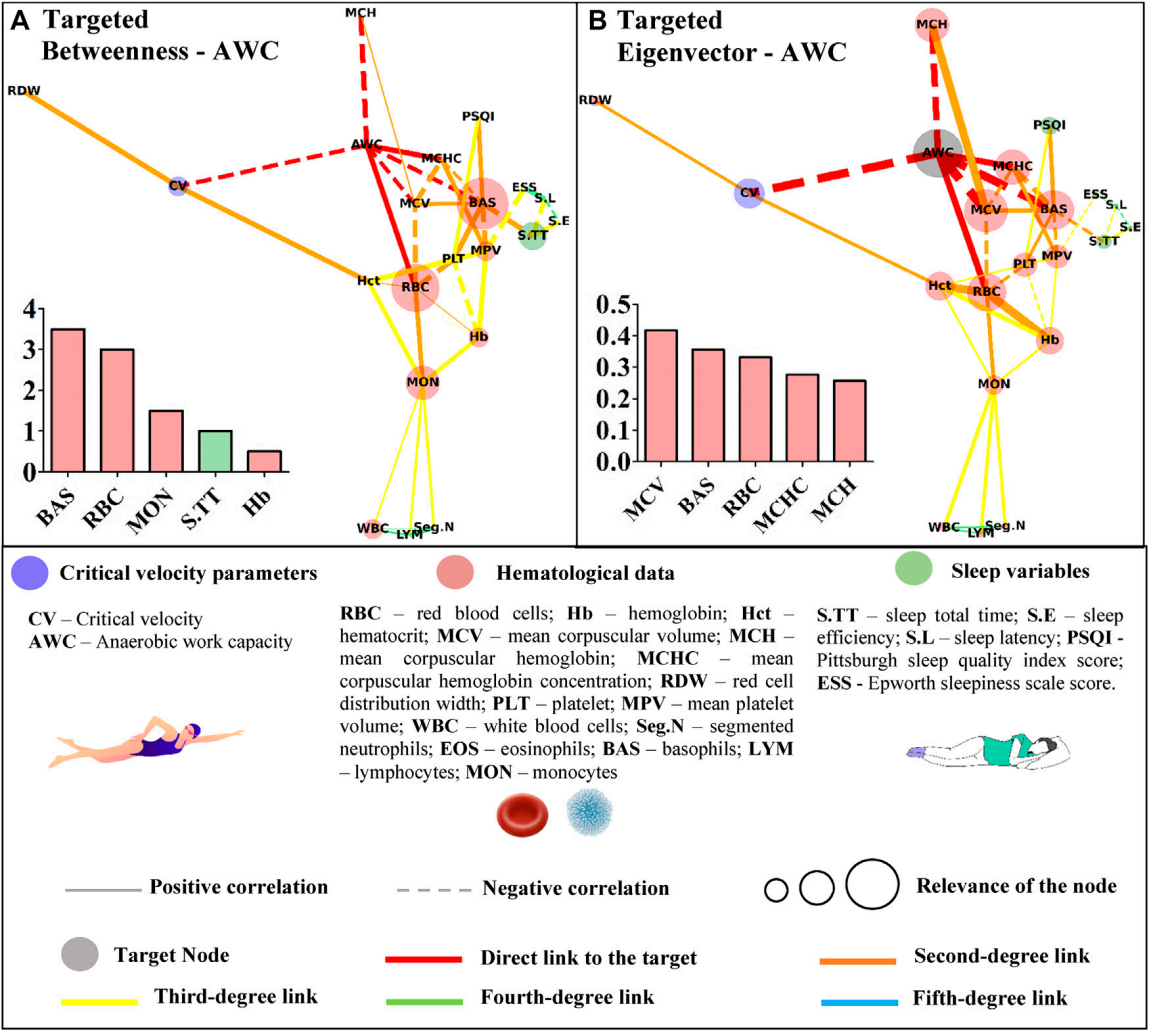
Centrality measurements from the targeted complex network model; **(A)** betweenness analysis considers the anerobic work capacity (AWC) as the target node. The thickness of the edge is directly related to the “distance” between the nodes connected by the edge (lower thickness means closer distances); **(B)** eigenvector analysis considers the anerobic work capacity (AWC) as the target node. The thickness of the edge is directly related to the strength of the edge connection (lower thickness means weaker connections).

Table 1 presents descriptive results from the critical velocity protocol, sleep questionnaires, and hematological analyses. Time to exhaustion increased as far as distances increased (*p* = 0.000), and high *R* ^2^ was obtained. Based on the correlation’s coefficients, the untargeted (Figure 3) and targeted (Figures 4, 5) complex networks were generated. Concerning the untargeted network, monocytes, basophils, mean platelet volume, hematocrit, red blood cells, and AWC appeared as the shortest paths to the other nodes (Figure 3A). Except for monocytes, the same variables along with hemoglobin and platelets were highlighted in the eigenvector centrality (Figure 3B).

**TABLE 1:** Outcomes from the critical velocity protocols, sleep questionnaires, and hematological analyses.

Critical velocity protocol	Mean ± SD	Range	CI
100-m (s)	73 ± 9	56–93	7–11
200-m (s)	170 ± 16	138–217	13–21
400-m (s)	354 ± 43	279–492	35–56
800-m (s)	747 ± 84	576–946	68–109
CV (m/s)	1.05 ± 0.11	0.80–1.35	0.09–0.14
AWC (m)	25 ± 7	12–42	6–9
*R* ^2^	0.999 ± 0.001	0.998–0.999	0.001–0.001
Sleep variables			
PSQI (score)	4.8 ± 1.3	3–10	1.1–1.7
Sleep total time (min)	563 ± 116	285–810	95–150
Sleep latency (min)	20 ± 17	5–90	14–22
Sleep efficiency (%)	93 ± 8	68–100	6–10
ESS (score)	8.3 ± 4.2	0–17	3.4–5.4
Hematological variables			
Red Blood Cells (10^6^/ul)	4.83 ± 0.37	4.13–5.56	0.30–0.48
Hemoglobin (g/dl)	13.8 ± 1.01	12.0–15.5	0.82–1.31
Hematocrit (%)	42.8 ± 2.95	37.3–48.1	2.41–3.82
Mean corpuscular volume (fl)	88.6 ± 3.40	76.5–94.5	2.77–4.40
Mean corpuscular hemoglobin (pg)	28.7 ± 1.04	25.3–30.3	0.85–1.35
Mean corpuscular hemoglobin concentration (10^6^/ul)	32.3 ± 0.5	31.3–33.4	0.4–0.6
Red cell distribution width (%)	13.4 ± 0.4	12.4–14.7	0.3–0.5
Platelet (10^9^/L)	262.8 ± 49.3	175–400	40.1–63.8
Mean platelet volume (fl)	8.7 ± 1.03	6.9–10.7	0.84–1.33
White Blood Cells (10^9^/ul)	6776 ± 1880	3500–11,000	1532–2432
Segmented neutrophils (10^9^/L)	3462 ± 1332	1390–6826	1085–1723
Eosinophils (10^9^/L)	198 ± 128	0–561	104–165
Basophils (10^9^/L)	17 ± 15	0–58	12–19
Lymphocytes (10^9^/L)	2597 ± 660	1579–4049	538–853
Monocytes (10^9^/L)	501 ± 169	270–870	137–218

CV, Critical velocity; AWC, Anaerobic work capacity; PSQI, Pittsburgh sleep quality index score; ESS, Epworth sleepiness scale score; SD, Standard deviation; CI, confidence interval.

Restrictedly to the network considering CV as the target, hematocrit, AWC, hemoglobin, basophils, and monocytes were highlighted in this metric (Figure 4A). Regarding the eigenvector, hematocrit appeared again as the most influential node, followed by segmented neutrophils, red blood cells, hemoglobin, and AWC (Figure 4B).

Basophils appeared as the most important path to reach AWC in the betweenness analysis. Other hematological variables (red blood cells, monocytes, and hemoglobin) along with sleep total time appeared in the sequence (Figure 5A). The eigenvector analysis showed that hematological variables (mean corpuscular volume, basophils, red blood cells, mean corpuscular hemoglobin concentration, and mean hemoglobin concentration) were the most influential for AWC (Figure 5B).

## Discussion

The complex network model adopted in this study provided new insights into the link between sleep and hematologic profile and their association with the parameters derived from the critical velocity protocol. While the untargeted approach offered a broad view of the relationship between the studied data, the targeted analysis revealed the most important parameters for the CV and AWC. To the best of our knowledge, the relationships presented by this targeted complex network are pioneers in the swimming science field.

Untargeted networks have revealed integrative and global visualization of complex structures, enabling further advances in distinct areas of knowledge (Strogatz, 2001; Palla et al., 2005), including physical exercise science (Pereira et al., 2015; Pereira et al., 2018; Cirino et al., 2021; Breda et al., 2022; Fiori et al., 2022). In this study, the untargeted approach offers insights for scientists to investigate, in future studies, the proper impact of the connections presented in the graph. The untargeted network is limited in deeply exploring the revealed associations, but without this analysis, such outcomes would remain unknown to swimming science. In the betweenness metric, for instance, hematological data have stood out. However, AWC has also appeared as a common path for the remaining data in the network, which is interesting given the debate regarding its physiological significance (Dotan, 2022). Hematological parameters were also highlighted as the most important nodes inside the eigenvector metric, but future studies are required to explore their impact on the other variables inserted in the untargeted network.

The higher scores for some hematological data in the untargeted approach may have resulted from the correlations between the hematological variables. This network has a systemic and not directed score that could indicate whether the bias introduced by the different classes of parameters was suppressed in the targeted approach. In this sense, the sleep total time of young swimmers was positively associated with sleep efficiency (r = 0.45; *p* = 0.004) and inversely correlated with sleep latency (and r = −42; *p* = 0.008). However, among these variables, only sleep total time was significantly associated with basophils (r = −0.33; *p* = 0.040), which in turn, appeared as an influential node in both centrality metrics of the networks with CV or AWC as targets (Figures 4, 5). Sleep loss affects the white blood cells and is associated with inflammatory processes (Simpson and Dinges, 2007). On this matter, Short et al. (2018) experimentally manipulated the sleep total time of adolescents and estimated 9.35 h to avoid sleep deficits. The young swimmers who were part of this study reported sleep duration close to this and other (Owens, 2006; Campbell et al., 2018) estimations, sustaining the inverse association between this result and basophils. Thus, by only looking at the direct associations, one may consider that sleep total time does not influence CV or AWC. However, the complex network allows suggesting that as far as sleep duration is adequate, sleep efficiency seems to positively modulate the latency (r = −0.67; *p* = 0.000), which in turn, may affect the basophils and, ultimately, AWC and CV.

Sleep quality (i.e., PSQI) did not appear as an influent node in the targeted networks. On the other hand, it is interesting to note that basophils and this variable were positively associated (r = 0.43; r = 0.006). In patients with distinct levels of obstructive sleep apnea, basophils and the apnea-hypopnea index were significantly correlated (Fan et al., 2019). On the other hand, none of the evaluated young swimmers reported sleep apnea. Moreover, only 23% of the young swimmers presented scores higher than the five cutoffs, indicating that most of our patients had good sleep quality (Buysse et al., 1989). Thus, although the PSQI global score is a reliable measure of sleep quality (Mollayeva et al., 2016), further studies are required not only to better comprehend its association with basophils but also to verify if the sleep quality provided by the PSQI may affect the targeted network topology described in this study.

Another interesting result is that RBC was highlighted in almost all target networks (except for the betweenness analysis with CV as the target). However, all direct connections of this parameter emerged from the red blood profile, and no relationship with sleep variables was observed. On the other hand, the excessive daytime sleepiness (i.e., ESS) was associated with PLT (r = 0.50; *p* = 0.001) and MPV (r = −0.34; *p* = 0.035), with the former directly linked to RBC (r = −0.33; *p* = 0.044). Literature has demonstrated the link between sleep pathologies and platelet count and volume (Alonso-Fernandez et al., 2020; Chang et al., 2020). On the other hand, we retake that none of the evaluated young swimmers reported any sleep disorder. Thus, it is possible to suggest, in a non-pathologic condition, that ESS affects PLT, which in turn is associated with RBC that is directly linked with AWC.

Apart from the sleep variables, both metrics identified hematocrit as the gatekeeper and the most influential node to reach CV. The ‘paradox of hematocrit’ is regularly debated by hemorheologists given its ambiguous aspect for oxygen supply (Brun et al., 2018). During exercise, the enhanced blood oxygen content may be functional when the hematocrit is suboptimal; thus, further circulatory functions should be accounted for the optimized oxygen transport (Boning et al., 2011). Accordingly, the targeted networks slightly advanced on this matter by demonstrating the overview of interactions between hematocrit and other hematological parameters, but most importantly, how they affect the network as a whole to reach CV. AWC also appeared as an important node inside betweenness and eigenvector metric developed to explore CV. Both parameters were inversely correlated (r = −0.42; *p* = 0.008), which agrees with the suggestion that these act in bioenergetic congruence during exercise (Poole et al., 2016). We are not aware of any published data relating red/white blood cells to these parameters, and our design cannot promote further insights into these relationships. However, the centrality metrics efficiently demonstrated some connection between these variables, and further experimental studies are required to advance on this matter.

The results of this study must be cautiously interpreted. The untargeted network provided a broad view of the connections between hematological, sleep, and physical performance data of young swimmers. Scientists from distinct areas can find valuable information in this analysis without focusing on the CV or AWC, for instance. Regarding the exercise physiology context, the associations revealed by the targeted network should not be considered to cause and effect. The targeted approaches were idealized for delivering personalized centrality scores which highlight the most important nodes to a selected target in the network. In this context, it is important to emphasize that such an analysis was not an arbitrary manipulation but yet obtained by pondering the network edges systematically or directing the destiny node.

Furthermore, evaluations comprised the third week of training after vacation. Thus, we cannot affirm that young swimmers had already huge physiological improvements; also, responsiveness to training is individual (Pickering and Kiely, 2019). Moreover, puberty timing and tempo vary substantially among adolescents (Roemmich and Rogol, 1995) and play an important role in circulating hormones (Varlinskaya et al., 2013; Livadas and Chrousos, 2016), with some affecting physical performance (Handelsman, 2017). Therefore, in light of these limitations, future studies should conduct further networks and explore if and how these factors may affect the described associations. Further designs involving psychological and nutritional data are also welcome to create a higher topological structure and demonstrate the interrelationship with the sleep and hematologic results, but ultimately, with CV and AWC.

## Conclusion

The untargeted approach revealed new connections among the studied data. This characterization paves the way for further research in an attempt to understand the proper relationship between hematological, sleep, and performance variables of young swimmers. In a more focused context, the Hct, AWC, Hb, BAS, and MON appeared as the shortest paths (i.e., betweenness metric) to link CV with the remaining variables. Some of these variables (Hb, BAS, and MON) were also short paths to reach AWC, but RBC and S.TT were also highlighted in this analysis. Although the betweenness metric revealed important paths to reach the nodes of interest, the eigenvector metric efficiently indicated those that may have a greater influence on CV and AWC. In this way, RBC seems to impact both aerobic and anerobic parameters. However, while Hct, Hb, MON, and the AWC primarily influenced CV, a distinct scenario was observed for AWC, which was affected by MCV, BAS, MCHC, and MCH. These results trigger new discussions surrounding the relevance of each highlighted parameter in the performance of young swimmers, which must be deeper explored in future studies.

## Data Availability

The raw data supporting the conclusions of this article will be made available by the authors, without undue reservation.
