# Supplementary material for: Associations among sleep, hematologic profile, and aerobic and anerobic capacity of young swimmers: A complex network approach
Source: Front Physiol. 2022 Aug 24;13:948422. doi: 10.3389/fphys.2022.948422 (PMC9448919; doi:10.3389/fphys.2022.948422)
Supplement: Supplementary file 1 [file DataSheet1.PDF]

Supplementary file

The following tables present every metric obtained in the complex network model. Figures 3, 4, and 5 inside the manuscript were generated by these data. To avoid long captions, we have provided a list of acronyms and their meanings.

CV – Critical velocity

AWC – Anaerobic work capacity

RBC – red blood cells;

Hb – hemoglobin;

Hct – hematocrit;

MCV – mean corpuscular volume;

MCH – mean corpuscular hemoglobin;

MCHC – mean corpuscular hemoglobin concentration;

RDW – red cell distribution width;

PLT – platelet;

MPV – mean platelet volume;

WBC – white blood cells;

Seg.N – segmented neutrophils;

EOS – eosinophils;

BAS – basophils;

LYM – lymphocytes;

MON – monocytes

S.TT – sleep total time;

S.E – sleep efficiency;

S.L – sleep latency;

PSQI - Pittsburgh sleep quality index score;

ESS - Epworth sleepiness scale score.

Supplementary Table 1. Centralities parameters for both target (critical velocity) and untargeted networks.

Node	Untarget Eigenvector	Eigenvector for the CV target	Untarget Betweenness	Betweenness for the CV target
AWC	8216.950872	8258.606255	8226.190476	20000
RBC	10536.66575	11991.908	8428.571429	0
MCV	7596.246026	3936.884795	3626.190476	0
MCH	3118.831742	2032.702185	0	0
MCHC	6937.792654	2532.740359	900	0
BAS	9704.411004	3532.720932	14233.33333	10000
CV	3767.806164	9872.901209	5950	0
Hb	9522.765306	11950.86057	4235.714286	20000
Hct	10143.47015	16831.0279	9935.714286	22500
PLT	10564.37241	6450.047776	5528.571429	0
MON	7379.150939	6494.117631	15300	7500
MPV	9658.553381	5170.431461	10919.04762	2500
S.TT	2231.96573	277.3062874	6733.333333	5000
PSQI	3997.618783	1105.54498	0	0
RDW	743.1268115	3007.497437	0	0
WBC	2403.538263	1387.731608	0	2500
Seg.N	2403.538263	1204.152708	0	0
LYM	2403.538263	1218.981697	0	0
ESS	2098.030216	407.2499661	3466.666667	0
S.L	978.9028519	31.24392095	1216.666667	0

Supplementary Table 2. Parameters used to construct the untargeted (coefficient and signal) and targeted (all parameters) complex network. In this case, the critical velocity is the target.

from	to	coefficient	signal	weight	relevance
AWC	RBC	0.333479	+	0.16674	0.5
AWC	MCV	0.482978	-	0.241489	0.5
AWC	MCH	0.332001	-	0.166	0.5
AWC	MCHC	0.321159	+	0.16058	0.5
AWC	BAS	0.441504	-	0.220752	0.5
CV	AWC	0.44611	-	0.44611	1
RBC	Hb	0.886363	+	0.221591	0.25
RBC	Hct	0.86709	+	0.433545	0.5
RBC	MCV	0.384868	-	0.096217	0.25
RBC	PLT	0.327805	-	0.081951	0.25
RBC	MON	0.408253	+	0.102063	0.25
MCV	MCH	0.827405	+	0.206851	0.25
MCV	MCHC	0.356744	-	0.089186	0.25
MCV	BAS	0.495809	+	0.123952	0.25
MCHC	MPV	0.410812	+	0.102703	0.25
MCHC	BAS	0.582637	-	0.145659	0.25
MPV	BAS	0.347493	-	0.086873	0.25
PLT	BAS	0.326163	+	0.081541	0.25
BAS	S.TT	0.334677	-	0.083669	0.25
BAS	PSQI	0.432753	+	0.108188	0.25
CV	Hct	0.353218	+	0.353218	1
CV	RDW	0.327492	+	0.327492	1
Hb	Hct	0.981095	+	0.490548	0.5
Hb	PLT	0.425402	-	0.10635	0.25
Hb	MPV	0.376811	+	0.094203	0.25
Hb	MON	0.469103	+	0.117276	0.25
Hct	PLT	0.39977	-	0.199885	0.5

Hct	MPV	0.323338	+	0.161669	0.5
Hct	MON	0.437633	+	0.218817	0.5
PLT	MPV	0.688648	-	0.172162	0.25
PLT	PSQI	0.500063	+	0.125016	0.25
WBC	MON	0.766119	+	0.19153	0.25
Seg.N	MON	0.656271	+	0.164068	0.25
LYM	MON	0.689391	+	0.172348	0.25
MPV	ESS	0.337636	-	0.084409	0.25
S.TT	S.L	0.422575	-	0.052822	0.125
S.TT	S.E	0.451235	+	0.056404	0.125
WBC	Seg.N	0.926546	+	0.115818	0.125
WBC	LYM	0.713262	+	0.089158	0.125
Seg.N	LYM	0.44887	+	0.056109	0.125
S.L	ESS	0.359072	+	0.044884	0.125
S.E	S.L	0.672168	-	0.042011	0.0625
RBC	AWC	0.333479	+	0.16674	0.5
MCV	AWC	0.482978	-	0.241489	0.5
MCH	AWC	0.332001	-	0.166	0.5
MCHC	AWC	0.321159	+	0.16058	0.5
BAS	AWC	0.441504	-	0.220752	0.5
AWC	CV	0.44611	-	0.44611	1
Hb	RBC	0.886363	+	0.221591	0.25
Hct	RBC	0.86709	+	0.433545	0.5
MCV	RBC	0.384868	-	0.096217	0.25
PLT	RBC	0.327805	-	0.081951	0.25
MON	RBC	0.408253	+	0.102063	0.25
MCH	MCV	0.827405	+	0.206851	0.25
MCHC	MCV	0.356744	-	0.089186	0.25
BAS	MCV	0.495809	+	0.123952	0.25
MPV	MCHC	0.410812	+	0.102703	0.25
BAS	MCHC	0.582637	-	0.145659	0.25
BAS	MPV	0.347493	-	0.086873	0.25
BAS	PLT	0.326163	+	0.081541	0.25

S.TT	BAS	0.334677	-	0.083669	0.25
PSQI	BAS	0.432753	+	0.108188	0.25
Hct	CV	0.353218	+	0.353218	1
RDW	CV	0.327492	+	0.327492	1
Hct	Hb	0.981095	+	0.490548	0.5
PLT	Hb	0.425402	-	0.10635	0.25
MPV	Hb	0.376811	+	0.094203	0.25
MON	Hb	0.469103	+	0.117276	0.25
PLT	Hct	0.39977	-	0.199885	0.5
MPV	Hct	0.323338	+	0.161669	0.5
MON	Hct	0.437633	+	0.218817	0.5
MPV	PLT	0.688648	-	0.172162	0.25
PSQI	PLT	0.500063	+	0.125016	0.25
MON	WBC	0.766119	+	0.19153	0.25
MON	Seg.N	0.656271	+	0.164068	0.25
MON	LYM	0.689391	+	0.172348	0.25
ESS	MPV	0.337636	-	0.084409	0.25
S.L	S.TT	0.422575	-	0.052822	0.125
S.E	S.TT	0.451235	+	0.056404	0.125
Seg.N	WBC	0.926546	+	0.115818	0.125
LYM	WBC	0.713262	+	0.089158	0.125
LYM	Seg.N	0.44887	+	0.056109	0.125
ESS	S.L	0.359072	+	0.044884	0.125
S.L	S.E	0.672168	-	0.042011	0.0625

Supplementary Table 3. Centralities parameters for both targeted (anaerobic work capacity) and untargeted networks.

Node	Untarget Eigenvector	Eigenvector for the AWC target	Untarget Betweenness	Betweenness for the AWC target
AWC	8216.950872	16182.69683	8226.190476	0
RBC	10536.66575	9985.330628	8428.571429	15000
MCV	7596.246026	12520.74733	3626.190476	0
MCH	3118.831742	7734.836099	0	0
MCHC	6937.792654	8313.045152	900	0
BAS	9704.411004	10736.8623	14233.33333	17500
CV	3767.806164	6102.71965	5950	2500
Hb	9522.765306	5036.289534	4235.714286	2500
Hct	10143.47015	5586.407135	9935.714286	0
PLT	10564.37241	3955.217124	5528.571429	0
MON	7379.150939	2519.986605	15300	7500
MPV	9658.553381	3811.711095	10919.04762	2500
S.TT	2231.96573	1335.863958	6733.333333	5000
PSQI	3997.618783	2065.310783	0	0
RDW	743.1268115	732.4718845	0	0
WBC	2403.538263	407.2095216	0	2500
Seg.N	2403.538263	352.4238363	0	0
LYM	2403.538263	359.4707835	0	0
ESS	2098.030216	239.7357121	3466.666667	0
S.E	633.2758546	117.7606432	0	0

Supplementary Table 4. Parameters used to construct the untargeted (coefficient and signal) and targeted (all parameters) complex network. In this case, the anaerobic work capacity is the target.

from	to	coefficient	signal	weight	relevance
AWC	RBC	0.333479	+	0.333479	1
AWC	MCV	0.482978	-	0.482978	1
AWC	MCH	0.332001	-	0.332001	1
AWC	MCHC	0.321159	+	0.321159	1
AWC	BAS	0.441504	-	0.441504	1
CV	AWC	0.44611	-	0.44611	1
RBC	Hb	0.886363	+	0.443181	0.5
RBC	Hct	0.86709	+	0.433545	0.5
RBC	MCV	0.384868	-	0.192434	0.5
RBC	PLT	0.327805	-	0.163903	0.5
RBC	MON	0.408253	+	0.204126	0.5
MCV	MCH	0.827405	+	0.413703	0.5
MCV	MCHC	0.356744	-	0.178372	0.5
MCV	BAS	0.495809	+	0.247905	0.5
MCHC	MPV	0.410812	+	0.205406	0.5
MCHC	BAS	0.582637	-	0.291319	0.5
MPV	BAS	0.347493	-	0.173747	0.5
PLT	BAS	0.326163	+	0.163081	0.5
BAS	S.TT	0.334677	-	0.167339	0.5
BAS	PSQI	0.432753	+	0.216376	0.5
CV	Hct	0.353218	+	0.176609	0.5
CV	RDW	0.327492	+	0.163746	0.5
Hb	Hct	0.981095	+	0.245274	0.25
Hb	PLT	0.425402	-	0.10635	0.25
Hb	MPV	0.376811	+	0.094203	0.25
Hb	MON	0.469103	+	0.117276	0.25
Hct	PLT	0.39977	-	0.099943	0.25

Hct	MPV	0.323338	+	0.080834	0.25
Hct	MON	0.437633	+	0.109408	0.25
PLT	MPV	0.688648	-	0.172162	0.25
PLT	PSQI	0.500063	+	0.125016	0.25
WBC	MON	0.766119	+	0.19153	0.25
Seg.N	MON	0.656271	+	0.164068	0.25
LYM	MON	0.689391	+	0.172348	0.25
MPV	ESS	0.337636	-	0.084409	0.25
S.TT	S.E	0.451235	+	0.112809	0.25
S.TT	S.L	0.422575	-	0.105644	0.25
WBC	Seg.N	0.926546	+	0.115818	0.125
WBC	LYM	0.713262	+	0.089158	0.125
Seg.N	LYM	0.44887	+	0.056109	0.125
S.L	ESS	0.359072	+	0.044884	0.125
S.E	S.L	0.672168	-	0.084021	0.125
